# Choosing a Surgeon: An Exploratory Study of Factors Influencing Selection of a Gender Affirmation Surgeon

**DOI:** 10.1089/trgh.2016.0011

**Published:** 2016-07-01

**Authors:** Randi Ettner, Frederic Ettner, Tonya White

**Affiliations:** ^1^ New Health Foundation Worldwide, Evanston, Illinois.; ^2^ Northwestern University, Evanston, Illinois.; ^3^ University of Chicago, Chicago, Illinois.; ^4^ Department of Child and Adolescent Psychiatry, Erasmus University Medical Centre, Rotterdam, The Netherlands.

**Keywords:** transgender, surgery, provider selection

## Abstract

**Purpose:** Selecting a healthcare provider is often a complicated process. Many factors appear to govern the decision as to how to select the provider in the patient–provider relationship. While the possibility of changing primary care physicians or specialists exists, decisions regarding surgeons are immutable once surgery has been performed. This study is an attempt to assess the importance attached to various factors involved in selecting a surgeon to perform gender affirmation surgery (GAS). It was hypothesized that owing to the intimate nature of the surgery, the expense typically involved, the emotional meaning attached to the surgery, and other variables, decisions regarding choice of surgeon for this procedure would involve factors other than those that inform more typical healthcare provider selection or surgeon selection for other plastic/reconstructive procedures.

**Methods:** Questionnaires were distributed to individuals who had undergone GAS and individuals who had undergone elective plastic surgery to assess decision-making.

**Results:** The results generally confirm previous findings regarding how patients select providers.

**Conclusion:** Choosing a surgeon to perform gender-affirming surgery is a challenging process, but patients are quite rational in their decision-making. Unlike prior studies, we did not find a preference for gender-concordant surgeons, even though the surgery involves the genital area. Providing strategies and resources for surgical selection can improve patient satisfaction.

## Introduction

Several studies have focused on the relative importance patients place on various factors when selecting healthcare providers. Hopkins et al. first reported that women preferred to see a female family physician if one was available.^1^ Several other early studies confirmed this finding.^2–6^ Other investigators, however, found the gender of the physician to be among the least important factors in physician selection.^7^ Schmittdiel et al. reported that female patients who chose female physicians were the least satisfied when patient satisfaction was assessed.^8^

In a study of 600 individuals, Bornstein et al. reported that variables relating to a physician's expertise, for example, board certification and professional skills, were most important in the choice of a primary care physician.^9^ Factors pertaining to office management issues ranked second in importance. Personal characteristics of the doctor, that is, gender, race, and religion, were rated as unimportant by the participants. The authors concluded that consumers are quite rational in the variables they deem important to their choice of a primary care doctor. Other studies likewise confirm the importance patients place on pragmatic concerns, such as convenience, and the negligible role of the doctor's personal characteristics in decision-making.^10,11^

It has been established that patients use different criteria when selecting a surgeon. Presumably, one anticipates an ongoing relationship with a primary care physician, but not with a surgical specialist. Not surprisingly, patients value a patient-centered approach—the creation of a therapeutic alliance—as more important in the relationship with the family physician or internist than with the surgical specialist, where technical expertise and experience are the most important determinants in the selection process.^12^

An exception occurs when the surgical specialist will perform procedures perceived as invasive, such as colonoscopy, or intimate—involving the anal/genital area—such as gynecological examinations. In these cases, both women and men prefer gender-concordant physicians. Women want female providers and men, to a lesser extent, prefer male physicians.^13–16^ Several investigators have looked at patient choice for selecting an obstetrician/gynecologist.^7,17–19^ These studies are particularly important to medical education and training as the number of men who apply to obstetrics/gynecology residency programs has steadily decreased. In 2005, only 24% of residents in this specialty were men compared with 53.5% in 1990 and 80.6% in 1978.^18^

In a well-designed study, 900 women were shown pictures of female and male obstetrician/gynecologists, without any descriptive information about the physicians. When no information about technical competence or caregiving qualities was provided, 83% of the participants chose the female as the preferred provider. When descriptors were provided, however, 62% of the women chose a male provider. The authors conclude that gender bias or stereotyping plays a role in patient choice.^18^ In 2006, Wolosin and Gesel investigated the assumption that proliferation of women entering the field of obstetrics and gynecology would humanize obstetrical practice and increase patient satisfaction.^20^ They were unable to confirm this. Instead, they concluded that physician gender had no impact on patient satisfaction.

## Purpose

The purpose of this study was to assess what factors are most important in patient selection of a surgeon to perform genital reconstruction. The choice of a surgeon for this procedure is often made from limited information and/or obtained indirectly from other patients or from marketing materials. Some aspects of technical expertise, such as whether the physician has been the target of malpractice claims, which credentials they possess, or specialized training pursued, may not be readily available. Many patients travel a great distance for surgery and therefore are unable to access information about staff, hospital facilities, or postoperative care venues beforehand.

Unlike those who undergo elective surgeries, persons who undergo gender affirmation surgery (GAS) have often longed for this procedure for decades. The intimate nature of the surgery, the profound emotional significance, and the out-of-pocket costs often involved are unique to those who undergo GAS. Additionally, there may be fears of stigmatization or discriminatory care from hospital personnel, stemming from prior experiences of healthcare disparities.

It was therefore hypothesized that patients' choice of a surgeon for GAS would be based on factors that differ from factors that determine patients' choice of a plastic or reconstructive surgeon. It was also hypothesized that contrary to anecdotal accounts, transwomen did not necessarily prefer to be treated by a surgeon who was herself a transwoman.

## Methods

Questionnaires were distributed to 54 transwomen who had undergone GAS and 47 cisgender patients who had undergone plastic or reconstructive surgery. All of the transwomen had met the World Professional Association for Transgender Health (WPATH) Standards of Care criteria for genital surgery. The participants were recruited locally from clinics where they received follow-up care. The cisgender comparison group was recruited from plastic surgery clinics and included those who had undergone elective (nongenital) surgical procedures. At the time of this investigation, no known transmen were performing GAS and therefore the participants were limited to transwomen.

The questionnaire was an adaptation of the Lerman Perceived Involvement in Care Scale.^21^ It included 22 questions that required rating the importance of 23 factors, using a 4-point scale, in determining the choice of a surgeon. They were also asked to rate the importance of those factors in the choice of a primary care physician.

The questionnaire encompassed the four domains of decision-making known to be most important with respect to choice of physicians: practical factors, information-based factors, personal characteristics of the physician, and emotional factors.^9^

Questions that asked participants to rate the importance of logistical factors, such as the location of a provider's office, financial issues (e.g., “The doctor was reasonably priced”; “The doctor accepted my insurance”), or ease of obtaining an appointment, were considered practical factors. Information-based factors assessed the patient's efforts to obtain facts about a physician's professional skills, such as number of complications, board certifications, and years of experience. Participants were asked to rate the importance of personal characteristics of the physician, which included age, gender, race, and ethnicity. Finally, a series of questions that evaluated emotional factors were incorporated into the questionnaire. These included questions asking to what extent one relied on gut feelings when choosing a provider, how important feeling comfortable with staff weighed in their choice of provider, and other similar queries.

## Results

Statistical analyses were performed using the R statistical package. Chi-square analyses were used for categorical variables and *t*-tests were used for continuous variables between groups.

### Demographic variables

The group of patients who had undergone GAS had a mean age of 49.0 years, ranging from 16 to 71 years of age. The plastic surgery group had a mean age of 55.2 years, ranging from 14 to 80 years of age. There was no difference between the groups with respect to education, race, ethnicity, and socioeconomic or employment status.

### Quantitative data

Patients in both groups rated the skills of the provider as the most salient feature of decision-making. Pragmatic concerns, such as a conveniently located facilities or ease of obtaining an appointment, were of secondary importance. There were no differences between groups with regard to the significance placed on age, gender, race, or ethnicity (personal characteristics) in the choice of the provider. Both groups viewed these factors as unimportant, contrary to the anecdotal accounts that transwomen would select surgeons who were themselves transgender, perceiving these surgeons to be more empathic.

There were some differences between groups. Participants with a history of GAS were more likely to choose a provider based on the recommendation of a trusted medical or mental health professional than the plastic surgery group. This was true in their choice of both primary care providers and surgeons (*p*<0.05). This group also relied heavily on information they found on the surgeon's website in decision-making (*p*=0.0001). Cost factored more heavily in the selection of a surgeon in this group (*p*=0.01) than in those undergoing elective cosmetic or reconstructive procedures.

Cisgender females who had undergone plastic surgery were more likely to consider the age of the primary care physician (*p*<0.05) and to listen to their gut feeling or intuition in choosing a primary care physician (*p*=0.006) than the group who underwent GAS. When choosing a surgeon for an elective procedure, they were more likely to have met face-to-face with a former surgical patient who had a good outcome (*p*=0.02).

### Qualitative data

Participants were given the option of commenting on any additional influences on their choice of primary care providers and surgeons. Several participants wrote comments indicating that emotional factors or personal characteristics of the provider entered into the decision-making process. One respondent wrote: “I wanted a primary care doctor who was involved with the LGBT community.” Another volunteered “I felt an instant connection with the surgeon…she went through transition.” Several respondents in both groups volunteered that they chose surgeons they knew socially.

## Discussion

Genital GASs, then referred to as sex reassignment surgeries, were first performed in the United States in 1966 when Johns Hopkins University Medical Center officially began its pioneer treatment program. By 1979, 20 major medical centers offered treatment, including surgery, to a select group of candidates—less than 10%—deemed appropriate.

Legal, religious, and medical critics mounted a challenge to surgical treatment for transsexualism. The media and the lay public echoed these concerns, endorsing the prevailing view that the condition was a manifestation of a psychiatric disorder. By 1979, Johns Hopkins stopped performing surgeries and most other centers followed suit.

Severely gender dysphoric individuals were left with few options. A number resorted to autocastration in the hope that a hospital would complete the surgery if the patient had the funds.^22^

Some found their way to Casablanca where Dr. Georges Burou, a French gynecologist, was performing vaginoplasty. Dr. Burou is credited with inventing the anteriorly pedicled penile skin flap inversion technique. Variations of this technique are still presently used. In 1969, Stanley Biber, a surgeon in Trinidad, Colorado, began performing vaginoplasties. The outstanding results achieved by Dr. Biber became widely known and patients flocked to him. He performed over 150 such operations annually. By 2000, he had performed over 4500 surgeries. In Europe, beginning in the 1970s, surgeries were being performed at the University Hospital of the Free University of Amsterdam, which became the leading center for the treatment of gender dysphoria.^23^

Needless to say, before the 1990s, obstacles to surgical treatment were daunting. As knowledge about gender dysphoria advanced and the internet provided greater access to information, more individuals sought surgery from the few practitioners who were willing to perform these surgeries.

Presently, the recognition that surgery is often medically indicated for gender dysphoria has been widely acknowledged and internationally endorsed. The World Professional Association for Transgender Health promulgates the Standards of Care, which set forth the criteria for provision of surgical treatment. In 2014, Medicare lifted a 30-year ban on its exclusion of genital surgery for gender dysphoria. Many insurance policies that previously excluded coverage now fund these procedures owing to the conclusive scientific evidence that surgery is not experimental, harmful, or cosmetic, but rather evidence-based best practice.

Thus, it is now possible to *choose* a surgeon, a previously inconceivable opportunity. Many qualified surgical specialists perform GAS worldwide. The surgical selection process in this unique situation has never been systematically explored. The present investigation is an attempt to shed light on this process.

Supporting the findings of previous research, the patients who participated in this investigation were quite rational in their strategy for choosing physicians and surgeons. Overall, patients used most, if not all the, means at their disposal to attain relevant information regarding providers' credentials, areas of specialization, outcomes, and experience.

The major limitation of this study is that only transwomen were queried and it does not take into account transmen, gender queer, and a comparison group of cisgender males. As more individuals are able to access surgery given the trend toward insurance coverage, future research will be needed to examine a larger group encompassing the spectrum of individuals requesting GAS.

Contrary to prior reports in the literature, there was no gender-concordant preference for surgeons among those undergoing genital reconstruction. Even though GAS is genital surgery, and therefore patients would presumably choose a female surgeon or a surgeon who herself had undergone GAS, this was not the case.

Patients who underwent GAS were less likely, as a group, to choose a primary care provider without doing due diligence. It is well known that transgender patients have often encountered prejudice from healthcare providers or received inadequate healthcare. It is therefore entirely predictable that given a choice, one would seek out providers reputed to be knowledgeable, respectful, and supportive of this population.

## Conclusion

The process of selecting a healthcare provider is challenging. Selection is often based on limited information, marketing materials, or information obtained indirectly. Yet, choosing a surgeon is a highly consequential decision. Understanding the factors that influence these decisions can be serviceable in creating resources to assist people in decision-making. Creating strategies that efficiently provide patients with the information that is relevant and serviceable would be empowering to patients and impact the quality of healthcare they receive.

## Abbreviation Used

GAS: gender affirmation surgery
